# Neurological manifestations in COVID‐19 caused by SARS‐CoV‐2

**DOI:** 10.1111/cns.13372

**Published:** 2020-04-07

**Authors:** Abdul Mannan Baig

**Affiliations:** ^1^ Department of Biological & Biomedical Sciences Aga Khan University Karachi Pakistan

## 1 COMMENT

**FIGURE 1:**
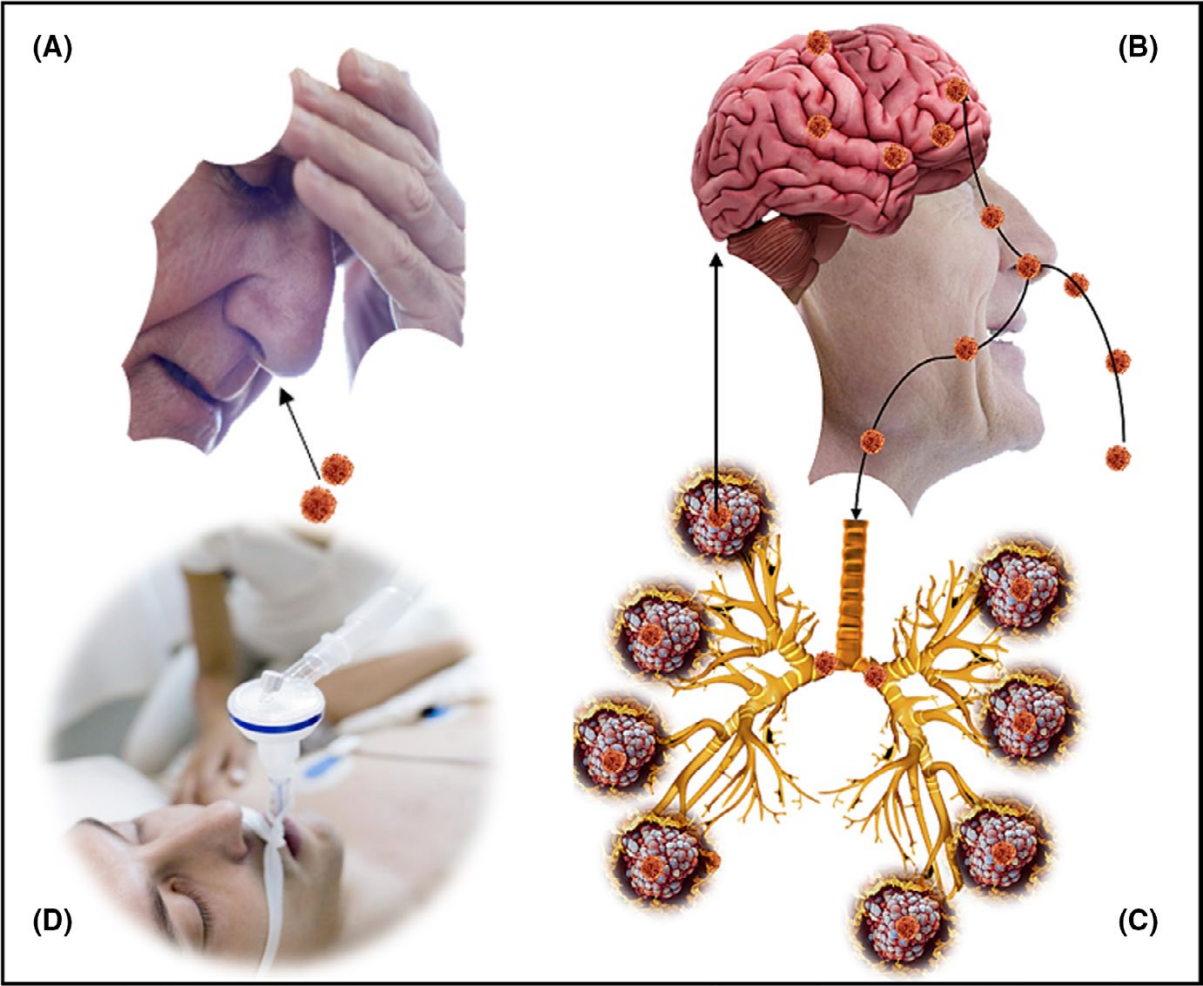
Neurological manifestations in COVID‐19. Fever with headaches (A) may occur early in COVID‐19 patients. Specific manifestations related to neurological deficits like loss of smell, taste, ataxia and convulsions have been reported in COVID‐19. The possible entry of SARS‐CoV‐2 to reach the brain via cribriform plate (B) or after systemic circulatory dissemination following infection of the lung (C), in early or late phases of COVID‐19 may result in loss of involuntary control of breathing resulting in acute respiratory insufficiency requiring assisted ventilation (D)

The recent outbreak of COVID‐19 caused by SARS‐CoV‐2 coronavirus has turned the world into chaos with its ominously high rate of transmissions. As the SARS‐CoV‐2 infection has become pandemic, the scientific community is in a race against time to beat the COVID‐19 by unraveling molecular targets and discover epitopes in the protein sequences of SARS‐CoV‐2 for vaccines/antibodies synthesis. It has been reported that in addition to the conventional respiratory complains of flu, patients are also exhibiting neurological signs and symptoms. Recently, the report of a patient with COVID‐19 exhibiting loss of the involuntary process of breathing^1^ controlled by the inspiratory area in the brainstem, is alarming. Additionally, neurological deficits reported in uncomplicated and complicated patients with COVID‐19^2^ from hospitals in Wuhan, China, are convincing enough that the neurological deficits could be ongoing in the recent outbreak without getting noticed. As the recent outbreak has now spread to almost all of the continents and has become pandemic, we are in the early phases of our attempts to understand the syndromic complexity of the COVID‐19. The SARS‐CoV‐2 causing COVID‐19 can take two pathways to involve the brain (Figure 1). Early occurrences of loss of smell, ataxia, and convulsions should be further evaluated for CNS involvement by SARS‐CoV‐2.

The clinicians throughout the world in general, and Wuhan, China, in particular, are getting the firsthand to study and report the real‐time clinical presentations of the patients affected by COVID‐19. The prognostic and diagnostic significance of neurological sign and symptoms in COVID‐19 patients can be gauged by fact that the protocol designed to investigate the First Few X cases (FFX) and their close contacts by the World Health Organization (WHO), includes a separate section for “other neurological signs” in addition to separate columns for respiratory symptoms.^3^ Additionally, reports of COVID‐19‐affected individuals experiencing convulsions in prevalent areas is alarming and need to be distinguished from febrile convolution that is expected to occur with high‐grade fever in patients with COVID‐19.

Our experience with taxonomically related SARS‐CoV patients in the past has proven beyond doubt the coronaviruses to affect the brain. Of many examples from the past, was a case where SARS‐CoV was isolated from the brain of a patient who had exhibited features of neurological deficits on 28th day of infection.^4^ In past outbreaks with SARS‐CoV, it has been shown that it targets the CNS^5^ and the reports that the brain also is an additional target of SARS‐CoV^4^ raises the possibility of the presentation of more patients with neurological manifestations in the ongoing outbreak of COIVD‐19. Also, SARS‐CoV has been isolated from brain tissue with edema and neuronal degeneration as seen at autopsies with immunohistochemistry, in situ hybridization, and electron microscopic confirmation of viral infection of the neurons.^6^ It would not be surprising to see the COVID‐19 virus following the same trend as both viruses are near identical taxonomically. As the pandemic is in effect at present, a detailed timeline of the syndromic neurological manifestation in COVID‐19 will emerge as more studies get published on complicated and uncomplicated cases of COVID‐19. Though the understanding of the pathogenetic mechanisms underlying the CNS invasion will be revealed in time, there is an urgent need to distinguish between neurologically affected CVOID‐19 patients and those who do not exhibit the sign and symptoms of CNS involvement. The hematogenous route appears to be the likely pathway for SARS‐CoV‐2 to reach the brain, but other routes to the CNS like across the cribriform plate (Figure 1B) of the ethmoid bone in proximity to the olfactory bulb^7^ should be taken into consideration in cases of early‐phase COVID‐19‐affected patients who exhibit loss of smell and taste accompanied with neurological signs and symptoms. Studies believe that direct SARS‐CoV infection of the human CNS does occur in some patients.^8^ It is also important to mention here that the neurological signs and symptoms observed in the COVID‐19 cases could be a manifestation of hypoxia, respiratory, and metabolic acidosis at an advanced stage of the disease, but reasonably, a differential diagnosis of these cases is needed, which could prove lifesaving. The later distinction also appears to be important from the vantage point of selecting a treatment regimen, as management of the COVID‐19 cases with neurological involvement would require more specific and aggressive treatments as compared to the patients without it.

The significance of a thorough neurological assessment of COVID‐19 patients cannot be overemphasized which can rule‐in or rule out a neurological deficit of a patient admitted after serological tests confirming the diagnosis of COVID‐19. Presence of neurological deficits followed by laboratory tests like serum urea, creatinine, electrolytes, and blood gases (PO_2_‐PCO_2_) can be helpful in the determination of primary or secondary involvement of the CNS in COVID‐19‐affected patients. With our experience and lessons learned from the past SARS‐CoV^4^, ^5^, ^6^ infections, specific investigation like attempts to isolate SARS‐CoV‐2 from CSF, as has been reported recently^9^, could prove to be of enormous advantage to diagnose an early or potentially complicated cases of COVID‐19. As neuronal death in CoV infections is not accompanied by substantial inflammation,^8^ clinical signs and symptoms of meningoencephalitis cannot be relied upon. As we are in a learning phase of what COVID‐19 presents with and how the patients are different from SARS‐CoV‐affected cases reported in the past, it is difficult if not impossible, to predict any particular diagnostic neurological test to ascertain the high‐risk COVID‐19 patients with covert infections of the CNS. As neurological deficits do occur in terminally ill patients with COVID‐19 (Figure 1D), an early differential diagnosis can be lifesaving in COVID‐19 patients. A biomarker in CSF or serum of the COVID‐19 patients with neurological deficits would have been of ideal to diagnose cases of COVID‐19 with CNS involvement, but with the unavailability of such methods, as of yet, smearing every possible method to include or exclude the COVID‐19 cases with neurological damage needs to be implemented.

We identified initially and stressed upon the inclusion of features like the loss of smell and taste that occur during the early phase of COVID‐19^7^ infections to be of significance. Attempts to isolate COVID‐19 from CSF^7^, ^9^ can be done in patients obvious findings of neurological involvement. With the vascular endothelium well known to express the ACE2 receptors (the target receptor of SARS‐CoV‐2), it would be interesting to see whether SARS‐CoV‐2 can be isolated from CNS at autopsy from the endothelial linings within the zones adjacent to the necrotic areas in COVID‐19 patients, as has been reported recently.^10^ The later study, and recent reports of detection of SARS‐CoV‐2 in the CSF of the COVID‐19 patient^9^ without a reasonable doubt validate our rationale of CNS being targeted by SARS‐CoV‐2 as pointed out recently^7^. Many news outlets, Blogs and COVID‐19 related information resources on Internet have helped in spreading our findings of “loss of smell and taste in COVID‐19” that has resulted in recognition of anosmia and hypogeusia as a significant alerting feature of COVID‐19. One example is that the American academy of otolaryngology‐head and neck surgery has also released a statement recently noting that anosmia and dysgeusia are ‘significant symptoms’ associated with COVID‐19^11^.

## References

[1] Li YC , Bai WZ , Hashikawa T . The neuroinvasive potential of SARS‐CoV2 may be at least partially responsible for the respiratory failure of COVID‐19 patients. J Med Virol. 2020 10.1002/jmv.25728 [Epub ahead of print] Review. PMC7228394 32104915

[2] Mao L , Wang M , Chen S , et al. Neurological Manifestations of Hospitalized Patients with COVID‐19 in Wuhan, China: a retrospective case series study. medRxiv. 10.1101/2020.02.22.20026500

[3] The World Health Organization (WHO) The First Few X (FFX) Cases and contact investigation protocol for 2019‐novel coronavirus (2019‐nCoV) infection. WHO REFERENCE NUMBER: WHO/2019‐nCoV/FFXprotocol/2020.2 CC BY‐NC‐SA 3.0 IGO; 29 January 2020 | Publication.

[4] Xu J , Zhong S , Liu J , et al. Detection of severe acute respiratory syndrome coronavirus in the brain: potential role of the chemokine mig in pathogenesis. Clin Infect Dis. 2005;41:1089‐1096. 16163626 10.1086/444461 PMC7107994

[5] Lau K , Yu W , Chu C , Lau S , Sheng B , Yuen K . Possible central nervous system infection by SARS coronavirus. Emerg Infect Dis. 2004;10(2):342‐344. 15030709 10.3201/eid1002.030638 PMC3322928

[6] Gu J , Korteweg C . Pathology and pathogenesis of severe acute respiratory syndrome. Am J Pathol. 2007;170(4):1136‐1147. 17392154 10.2353/ajpath.2007.061088 PMC1829448

[7] Baig AM , Khan NA . Novel chemotherapeutic strategies in the management of primary amoebic meningoencephalitis due to *Naegleria fowleri* . CNS Neurosci Ther. 2014;20(3):289‐290. 24456292 10.1111/cns.12225 PMC6493040

[8] Netland J , Meyerholz DK , Moore S , Cassell M , Perlman S . Severe acute respiratory syndrome coronavirus infection causes neuronal death in the absence of encephalitis in mice transgenic for human ACE2. J Virol. 2008;82(15):7264‐7275. 18495771 10.1128/JVI.00737-08 PMC2493326

[9] Zhou L , Zhang M , Gao J , Wang J . Sars‐Cov‐2: Underestimated damage to nervous system [published online ahead of print, 2020 Mar 24]. Travel Med Infect Dis. 2020; 10.1016/j.tmaid.2020.101642 PMC7269702 32220634

[10] Poyiadji N , Shahin G , Noujaim D , Stone M , Patel S , Griffith B . COVID-19-associated Acute Hemorrhagic Necrotizing Encephalopathy: CT and MRI Features [published online ahead of print, 2020 Mar 31]. Radiology. 2020; 10.1148/radiol.2020201187 PMC7233386 32228363

[11] ANOSMIA: NEW! COVID‐19 ANOSMIA REPORTING TOOL OPEN TO ALL CLINICIANS, American Academy of Otolaryngology‐ Head and Neck Surgery https://www.entnet.org/content/coronavirus-disease-2019-resources Accessed April 1, 2020.

